# Epidermal growth factor receptor in breast carcinoma: association between gene copy number and mutations

**DOI:** 10.1186/1746-1596-6-118

**Published:** 2011-12-02

**Authors:** Ning Lv, Xiaoming Xie, Qidong Ge, Suxia Lin, Xi Wang, Yanan Kong, Hongliu Shi, Xinhua Xie, Weidong Wei

**Affiliations:** 1 Department of Breast Oncology, Sun Yat-Sen University Cancer Center, Guangzhou, Guangdong 510060, P. R. China; 2 State Key Laboratory of Oncology in South China, Guangzhou, Guangdong 510060, P. R. China; 3 Department of Pathology, Sun Yat-Sen University Cancer Center, Guangzhou, Guangdong 510060, P. R. China

**Keywords:** breast cancer, epidermal growth factor receptor, EGFR, FISH, gene amplification, gene mutation, real-time PCR.

## Abstract

**Background:**

The epidermal growth factor receptor (EGFR) is an available target of effective anti-EGFR therapy for human breast cancer. The aim of this study was to assess the presence of EGFR gene amplification and mutations in breast cancer and to analyze the association between the statuses of these two gene alterations.

**Materials and methods:**

EGFR gene amplification and mutations were investigated in formalin-fixed, paraffin-embedded tissues from 139 Chinese female patients with breast cancer by means of fluorescence in-situ hybridization (FISH) and fluorescently labeled real-time quantitative polymerase chain reaction (RT-PCR), respectively.

**Results:**

EGFR gene amplification was observed in 46/139 (33.1%) of cases by FISH. Based on RT-PCR, 2/139 (1.4%) samples had EGFR gene mutations. Overall, only 1 (0.7%) of the cases was identified with both whole gene amplification and mutation, and 92 (66.2%) of cases were negative for both. High gene copy numbers of EGFR had significant correlation with the occurrence of EGFR protein expressions (P = 0.002).

**Conclusion:**

In this study, EGFR mutations were presented in only two samples, indicating that EGFR mutations should not be employed in future trials with anti-EGFR therapies for breast cancer. However, EGFR whole gene amplification is frequently observed in patients with breast cancer. It will be of significant interest to investigate whether EGFR gene copy number is a suitable screening test for EGFR-targeted therapy for breast cancer.

## Introduction

The human epidermal growth factor receptor (HER/EGFR/ErbB) family of receptor tyrosine kinases is comprised of four transmembrane growth factor receptor proteins that share similarities in structure and function. The epidermal growth factor receptor (HER-1/EGFR/ErbB1), encoded by the gene located on the short arm of chromosome 7, is a member of this family of Type I transmembrane tyrosine kinase receptors. EGFR is a 170 kDa transmembrane protein consisting of an intracellular domain (tyrosine kinase domain), a short transmembrane and juxtamembrane domain, and an extracellular domain (ligand-binding domain) with ligand-activated tyrosine kinase activity [1]. EGFR can be activated by various growth factor ligands, including epidermal growth factor (EGF) and the transforming growth factor-alpha (TGF-α). Ligand binding to EGFR results in homo- or hetero-dimerization of EGFR with another EGFR molecule or a different member of the ErbB family (e.g., HER2). This is followed by phosphorylation of the tyrosine kinase residue, which in turn induces the actual downstream signaling cascade [2-4]. Ligand-dependent activation of the EGFR tyrosine kinase residues serve as binding sites of signal transducer and activator proteins that mediate the downstream signaling processes of intracellular substrates [5]. The phosphatidyl inositol 3' kinase (PI3K) and Akt pathway and Ras/MAPK pathway are major signaling mechanisms, and they function in the control of several important biologic processes, including cell proliferation, survival, angiogenesis, and migration as well as resistance to apoptosis [6-8].

Due to the biologic significance of EGFR molecular signaling in carcinomas, several monoclonal antibodies against the ligand-binding domain of EGFR and small molecule tyrosine kinase inhibitors of the tyrosine kinase domain of EGFR have been investigated in the therapy of malignant tumors (e.g., non-small cell lung cancer [NSCLC], colorectal cancer [CRC] and metastatic breast cancer [MBC]) [9-16]. It is important to study whether EGFR is overexpressed in patients with breast cancer since these patients can be given specific EGFR molecule tyrosine kinase inhibitors such as gefitinib and lapatinib [15,16]. There are only a few reports regarding the overexpression of EGFR, with these studies indicating 8-36% of breast cancers over express this protein. However, systematic studies appraising EGFR gene amplification and mutations in the same set of cases among Chinese female patients with breast cancer are absent [17-19]. Many studies have concentrated on lung cancers, where most patients ultimately have a relapse. Mechanisms involved in resistance to targeted inhibition of lung cancer include secondary resistance mutations, inactivation of PTEN, activation of the MET pathway, minor clones with KRAS mutations, and adenocarcinoma transformation [20-32]. However, the mechanism of drug resistance in breast cancer is unknown.

The purpose of the present study was to examine 139 formalin-fixed, paraffin-embedded specimens from Chinese female patients with breast cancer, with a particular focus on the presence of EGFR gene amplification and mutations. We attempted to explore the relationship between EGFR copy numbers and EGFR mutations. We also analyzed the correlation between EGFR gene status and HER2 protein, estrogen receptor (ER), progesterone receptor (PR), and cytokeratin 5/6 (CK5/6) expression as well as Ki-67 index proliferation and intrinsic subtypes in these cases. In this study, we analyzed EGFR gene copy numbers by fluorescence in-situ hybridization (FISH), and the mutations were analyzed using a real-time (RT)-PCR detection kit.

## Methods and Materials

### Patient Information

One hundred and thirty-nine Chinese female patients with breast cancer who underwent surgery at the Department of Breast Oncology, Sun Yat-Sen University Cancer Center, from Jan 2010 to May 2011 were selected. The cases with primary breast cancer were randomly selected from the archives of our Department of Pathology based on the availability of blocks and sufficient tissues. Additionally, only cases with available EGFR FISH results, mutational status, and immunostaining were analyzed. Clinical information included age, disease stage, tumor type, mass size, and axillary lymph node metastasis status. The age interval of the patients was 25-75 years, with a mean of 50.8 years. The TNM Cancer Staging Manual 7th edition of the American Joint Committee on Cancer (AJCC) [33] was used to classify the cancer staging: stage 0, 2 cases; stage I, 28 cases; stage II, 78 cases; stage III, 30 cases; and stage IV, 1 case. Using immunohistochemistry as a surrogate definition of intrinsic subtypes for expression profiling, cases that were ER and/or PR positive, HER2 negative, and Ki-67 low (< 14%) were classified as luminal A cancers; cases that were ER and/or PR positive, HER2 negative, and Ki-67 high were classified as luminal B (HER2 positive) cancers; cases that were ER and/or PR positive, any Ki-67, and HER2 overexpressed or amplified were classified as luminal B (HER2 positive); cases that were HER2 overexpressed or amplified and ER and PR absent were classified as HER2 positive (non-luminal) cancers; cases that were ER and PR absent, HER2 negative, and CK5/6 and/or EGFR positive were classified as basal-like cancers; and cases that lacked expression of ER, PR, HER2, CK5/6, and EGFR were considered unclassified [34-36]. The histopathological classification of breast cancer in these cases was performed by an experienced pathologist of our pathology department. Ethics approval was obtained from the Ethical Review Committee of Sun Yat-Sen University Cancer Center, and informed consent was obtained from all patients.

### Tissue Preparation

Tissue microarrays were constructed using 0.6-mm tissue cores as previously described [37]. One core from the central and the other from the peripheral part of the tumor were sampled for each tumor. For each case, 4-5 μm sections of formalin-fixed and paraffin-embedded tissues were stained with Hematoxylin and Eosin for the establishment of the histopathological tumor type and differentiation grade.

### Fluorescence In-Situ Hybridization Analysis of EGFR Gene Copy Number

FISH analysis for EGFR gene copy number was performed according to the manufacturer's protocol using the GLP EGFR/CSP 7 probe (GP Medical Technologies, Beijing, China). Simply, the tissue microarray sections were incubated at 65°C overnight. The slides were deparaffinized in dimethyl benzene at room temperature for 10 minutes and dehydrated in 100% ethanol. After incubation in 30% sodium bisulfite at 50°C for 20-30 minutes, the sections were incubated in 2× saline sodium citrate buffer (2× SSC; pH 7.0) at 75°C for 5 minutes. The smears were digested with proteinase K (0.20 mg/ml in 2× SSC; pH 7.0) for 20-30 minutes at 37°C, followed by a rinse in 2× SSC at room temperature for 5 minutes and then dehydration in 70%, 85%, and 100% ethanol solutions in sequence. The solvents were changed frequently and regularly so that all traces of residual paraffin were removed in the above processes [38]. After amplification of the GLP EGFR/CSP 7 probe set, the slide was covered with a coverslip and sealed with Indian rubber. The slides were heated at 80°C for 8-10 minutes and were then hybridized at 42°C overnight. After a post-hybridization wash and dehydration of the samples, 4', 6'-diamidino-2-phenylindole (DAPI) was applied for chromatin counterstaining. At least 100 nuclei were scored for both EGFR gene signals and chromosome 7 signals under a magnification of 1000×.

The ratio was defined as: the total number of red signals (EGFR copy number) divided by the number of green signals (chromosome 7 copy number) in 100 nuclei. Tumors were scored as EGFR amplified when the EGFR FISH-positive results were: a) ratio ≥2.0, b) ≥15 copies of the red signals per cell in ≥10% of total cells, or c) the presence of EGFR gene clusters; or scored as EGFR polysomy when: ratio < 2.0, but ≥40% of cells display ≥4 copies. Meanwhile, EGFR FISH-negative include disomy (≥90% of cells display ≤2 copies), low trisomy (≥40% of cells display ≤2 copies, 10-40% of cells display 3 copies, and < 10% of cells display ≥4 copies), high trisomy (≥40% of cells display ≤2 copies, ≥40% of cells display 3 copies, and < 10% of cells display ≥4 copies), and low polysomy (10-40% of cells display ≥4 copies) [38-40]. Healthy cells were used as controls.

### Fluorescent PCR Method for Analysis of EGFR Gene Mutations

The 5-μm formalin-fixed and paraffin-embedded tissues were assayed for the presence of the most common EGFR mutations in exon 19 (E746-A750 and L747-P753insS short in-frame deletions) and exon 21 (L858R and L861Q point mutations) using a RT-PCR detection kit with the Taqman probe technique (GP Medical Technologies, Beijing, China). Genomic DNA was extracted from tissues using a TIANamp Genomic DNA kit (Tiangen Biotech, Beijing, China). Two of the exon 19 deletions and the exon 21 mutations of the EGFR gene were analyzed using fluorescently labeled RT-PCR products.

Amplification reactions were setup using reagents included in the Real Time PCR Detection Kit (GP Medical Technologies, Beijing, China), in accordance with the manufacturer's protocol. Essentially, the exon 19 PCR reaction consisted of 5.4 ul deionized water, 7.5 ul 2 × PCR pre-mix, 0.15 ul forward primers-1, 0.15 ul reverse primers-1, 0.15 ul probe-1 (delE746-A750), and 0.15 ul probe-2 (delL747-P753insS) in a total volume of 13.5 ul. The exon 21 PCR reaction consisted of 5.3 ul deionized water, 7.5 ul 2 × PCR pre-mix, 0.15 ul forward primers-2, 0.15 ul reverse primers-2, 0.2 ul probe-3 (L858R), and 0.2 ul probe-4 (L861Q) in a total volume of 13.5 ul. The PCR cycling program was as follows: 50°C for 2 min, 95°C for 10 min, and 40 cycles of 95°C for 15 sec, 62°C for 1 min. PCR analysis was performed by using an ABI Prism 7500 Real-Time PCR equipment (Applied Biosystems, Foster City, CA, USA) as previously described [41,42].

### Immunohistochemistry of EGFR Protein Expression

Immunohistochemical staining for EGFR was performed on the 5-μm formalin-fixed, paraffin-embedded tissue slide. EGFR was stained using the pharmDx kit according to manufacturer's instructions (DAKO). EGFR was scored positive if any membranous tumor cell staining was observed, whether or not it was completely circumferential. Staining intensity was scored as follows: 0, no membrane staining; 1+, weak staining intensity; 2+, moderate staining intensity; 3+, strong staining intensity. The staining intensity was multiplied by the percentage of tumor cells be stained to obtain a total score, resulting in a possible range 0 to 300. Samples with an overall score of 200 and higher were considered positive for EGFR-overexpression.

### Statistical analysis

SPSS version 13.0 software (SPSS, Chicago, IL, USA) was used to analyze the data. Associations between EGFR gene amplification and protein expression were evaluated using Pearson's chi-square test with cross tables. Differences of P < 0.05 were considered significant. Pearson's Chi-squared test or Fisher's exact test was also applied for evaluation of multiple comparisons between EGFR amplification and expression and age, disease stage, tumor type, axillary lymph node metastasis status, and immunohistochemical index (i.e., ER, PR, and HER2). A P value < 0.05 was considered statistically significant.

## Results

### EGFR Gene Copy Numbers in Breast Carcinomas

**Figure 1:**
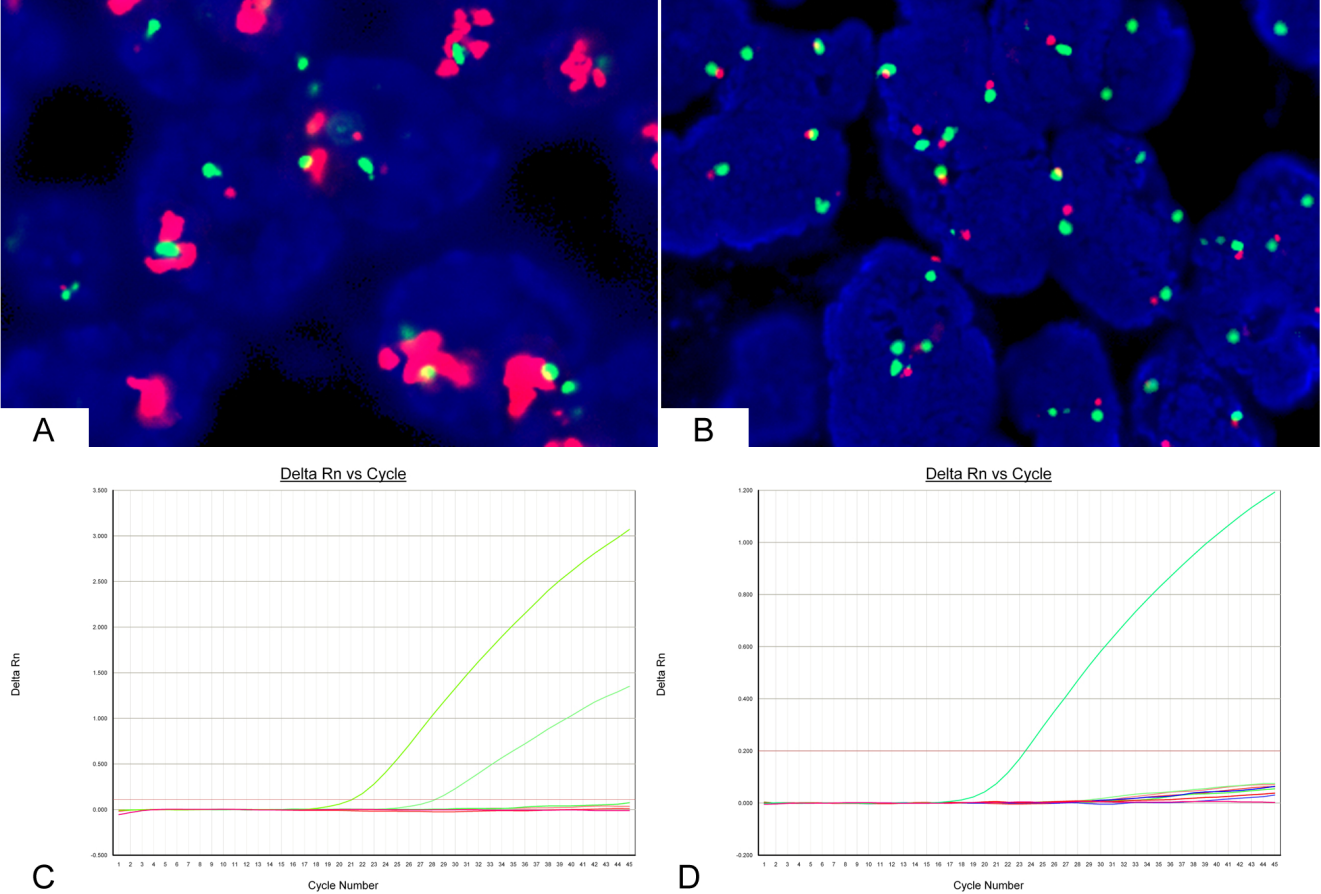
**Analysis of EGFR gene amplification and mutations based on FISH and RT-PCR analysis**. A: FISH positive (amplification), B: FISH negative (low trisomy), C: mutation positive (the left ascending curve represents the positive control, the right ascending curve represents the exon 21 L858R mutation), D: mutation negative (the ascending curve represents the positive control).

We obtained both FISH and RT-PCR EGFR data on 139 female patients with breast cancer. A total of 48 (34.5%) of the 139 tumors presented EGFR disomy, 6 tumors (4.3%) presented low trisomy, 3 tumors (2.2%) presented high trisomy, 36 (25.9%) tumors presented low polysomy, 42 tumors (30.2%) presented high polysomy, and 4 tumors (2.9%) presented amplification. From the total of 139 tumors, 46 (33.1%) presented positivity with FISH; 93 (66.9%) did not demonstrate EGFR gene amplification (Figure 1).

### EGFR Gene Mutations in Breast Carcinomas

A total of 2 (1.4%) of the 139 tumors harbored EGFR mutations, which include 1 exon 19 deletion with low trisomy of the EGFR gene copy number and 1 exon 21 L858R mutation with high polysomy of the EGFR gene copy number. None of the cases presented both of these mutations. Overall, one tumor was poorly prognostic triple-negative moderate-grade invasive ductal carcinoma; the other was also a moderate-grade invasive ductal carcinoma and ER positive. Both of the patients were postmenopausal with pathological stage IIA and negative for axillary lymph node metastasis.

### EGFR Protein Expression in Breast Carcinomas

**Figure 2:**
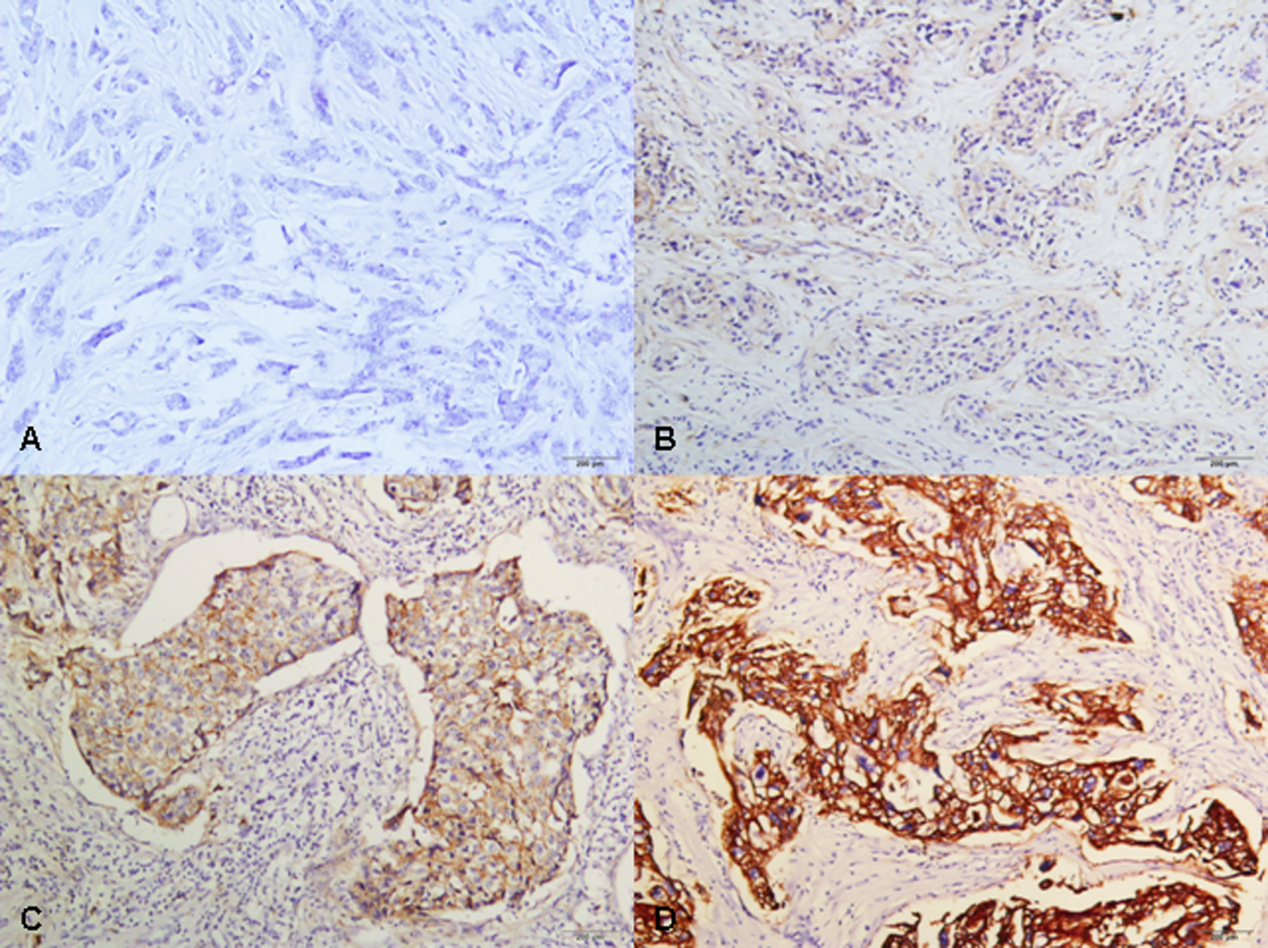
**EGFR protein expression by immunohistochemistry**. A: Negative EGFR expression; B: 1+ EGFR expression; C: 2+ EGFR expression; D: 3+ EGFR expression.

Based on immunohistochemistry, twenty-five (18.0%) of the 139 cases had an immunohistochemical score of 200 or more; the remaining 114 (82.0%) cases showed a negative result (Figure 2).

### Association between the EGFR and Clinical and Pathologic Features

The clinical and pathological features of all cases were evaluated for the purpose of determining clinically relevant correlations. EGFR gene copy number was associated with Ki-67 proliferation index (P = 0.007). EGFR FISH positivity was not associated with clinical-pathological features, including age (P = 0.265), lymph node metastasis (P = 0.765), disease stage (P = 0.748), tumor type (P = 0.551), ER status (P = 0.464), PR status (P = 0.943), and HER2 status (P = 0.733). The invasive ductal carcinomas group showed a trend toward higher EGFR gene amplification, although the association with tumor type was not statistically significant. EGFR protein expression was associated with molecular subtypes (luminal subtypes vs. others, P = 0.010), grade (II vs. III, P = 0.001), ER (P = 0.002), and PR (P < 0.001), respectively (Table 1).

**Table 1:** Summary of the relationship between EGFR copy number, expression, and the patients' clinical-pathological characteristics

clinical- pathological characteristic	Copy number			Expression		
	
	Positive, N/%	Negative, N/%	P value	Positive, N/%	Negative, N/%	P value
Age(years)						
≤35	5/10.9	4/4.3	0.265^c^	23/92.0	107/93.9	1.000^c^
>35	41/89.1	89/95.7		2/8.0	7/6.1	
Lymph node metastasis						
Positive	23/50	49/52.7	0.765^a^	16/64.0	56/49.1	0.178^a^
Negative	23/50	44/47.3		9/36.0	58/50.9	
Stage						
0	0/0	2/2.2	0.748^a, $^	1/4.0	2/1.8	0.450^a, $^
I	10/21.7	18/19.4		6/24.0	21/18.4	
II	25/54.3	53/57		11/44.0	67/58.8	
III	10/21.7	20/21.5		7/28.0	22/20.2	
IV	1/2.2	0/0		0/0	1/0.9	
Tumor type						
DCIS	0/0	3/3.2	0.551^b, &^	1/4.0	2/1.8	1.000^c, &^
LCIS	0/0	0/0		0/0	0/0	
IDC	44/95.7	83/89.2		23/92.0	104/91.2	
ILC	0/0	3/3.2		0/0	3/2.6	
Other	2/4.3	4/4.3		1/4.0	5/4.4	
ER						
Positive	33/71.7	72/77.4	0.464^a^	13/52.0	92/80.7	0.002^a^
Negative	13/28.3	21/22.6		12/48.0	22/19.3	
PR						
Positive	27/58.7	54/58.1	0.943^a^	5/20.0	76/66.7	0.000^a^
Negative	19/41.3	39/41.9		20/80.0	38/33.3	
HER2						
Positive	9/19.6	16/17.2	0.733^a^	5/20.0	29/25.4	0.567^a^
Negative	37/80.4	77/82.8		20/80.0	85/74.6	
Ki-67 (%)						
< 14	7/15.2	35/37.6	0.007^a^	17/68.0	67/58.8	0.393^a^
> 16	39/84.8	58/62.4		8/32.0	47/41.2	
Subtypes						
LUMA	9/19.6	28/30.1	0.628^a^, ^	4/16.0	33/28.9	0.010^c^, ^
LUMB (HER2-NEG)	17/37.0	32/34.4		7/28.0	42/36.8	
LUMB (HER2-POS)	10/21.7	16/17.2		4/16.0	22/19.3	
HER2	2/4.3	6/17.2		1/4.0	7/6.1	
Basal-like	6/13.0	4/4.3		9/36.0	1/0.9	
UC	2/4.3	7/7.5		0/0	9/7.9	
Grade						
I	0/0	0/0	0.921^a, #^	0/0	0/0	0.001^a, #^
II	31/67.4	52/55.9		10/40.0	73/64.0	
III	12/26.1	21/22.6		13/52.0	20/17.5	
UC	3/6.5	20/21.5		2/8.0	21/18.4	

^a^P values (two-sided) calculated using Pearson's chi-square test.

^b^P values (two-sided) calculated using Fisher's exact test.

^c^P values (two-sided) calculated using Continuity Correction of Pearson's chi-square test.

^$^Pearson's chi-square test for stage 0-II and III- IV vs. EGFR status.

^&^Fisher's exact test for invasive ductal carcinoma and other types vs. EGFR status.

^Pearson's chi-square test for luminal subtypes and other subtypes vs. EGFR status.

^#^Grade II and III vs. EGFR status.

DCIS, ductal carcinoma in-situ; LCIS, lobular carcinoma in-situ; IDC, invasive ductal carcinoma; ILC, invasive lobular carcinoma; ER, estrogen receptor; PR, progesterone receptor; HER2, human epidermal growth factor receptor-2; Ki-67, Ki-67 proliferation index; LUMA, luminal A; LUMB (HER2-NEG), luminal B (HER2-negative); LUMB (HER2-POS), luminal B (HER2-positive); UC, unclassified.

### Association of EGFR Protein Expression and EGFR Gene Amplification

15 of the 46 breast carcinomas with EGFR FISH positivity showed immunohistochemical positive scores and 31 cases had negative results. There was a correlation between protein overexpression and gene amplification, the FISH-positive rate was significantly higher in the IHC-positive group than in the IHC-negative group (P = 0.002) (Table 2).

**Table 2:** Correlation of EGFR gene amplification and protein expression

Expression	Amplification		P value
	
	Positive	Negative	
Positive	15/32.6	10/10.8	0.002^$^
Negative	31/67.4	83/89.2	

^$ ^P values (two-sided) calculated using Pearson's chi-square test.

## Discussion

The aim of this study was to evaluate the frequency of EGFR gene amplification and mutations in 139 female patients with breast cancer. It is known that EGFR gene amplification indicates EGF-sensitive breast cancer. In one study, EGFR gene amplification and/or high EGFR expression were demonstrated as biological predictors of poor prognosis in breast carcinoma [43]. Due to the availability and benefit of anti-EGFR therapies, including both monoclonal antibodies (MoAbs) and small molecule tyrosine kinase inhibitors (TKI), for the treatment of various solid malignant tumors, such as non-small cell lung cancer (NSCLC), squamous cell carcinoma of the head and neck (HNSCC), and colorectal cancer (CRC), the role of EGFR gene status has been investigated in a number of clinical studies. Several data sets regarding EGFR gene amplification in breast cancer are accessible. In various trials, EGFR gene amplification in breast carcinomas was different, ranging between 0.8-28 percent. Khawla Al-Kuraya et al. [44] described EGFR gene amplification in 0.8% of studied tumors, Jungsil Ro et al. [43] reported positivity in 3 of the 21 evaluable cases, Rohit Bhargava et al. [45] found positive EGFR amplification in 11/175 (6%) of samples, Christian Kersting et al. [46] showed EGFR whole gene amplification in 4.7% of investigated cases, and in the cohorts of Judith A. Gilbert et al. [47] and Jorge S Reis-Filho et al. [48] 26% and 28% of the metastatic breast carcinomas displayed high EGFR copy number, respectively. It is likely that multiple techniques and scoring systems used in the detection of EGFR amplification have led to the inconsistent outcomes of these different trials.

In this current study we used FISH to detect EGFR gene copy numbers in breast carcinomas. We identified EGFR gene amplification in 46 (33.1%) of the 139 patients with breast cancer. This percentage is higher than the range reported by the studies mentioned above. It seems that the Chinese origin of the specimens, possibly in addition to the use of various techniques and scoring criteria, may possibly have contributed to the difference in the results. In this study, 46 carcinomas showed EGFR gene amplification, which include 42 tumors (30.2%) presenting high polysomy and 4 tumors (2.9%) presenting amplification (based on the ratio of EGFR gene copies to CEP7 gene copies in at least 100 tumor cell nuclei). These data revealed that EGFR gene amplification is a frequent event in Chinese patients with breast carcinomas. As observed from this study, EGFR positive immunostaining was consistent with EGFR gene amplification. It appears that positive EGFR protein overexpression could predict gene amplification in breast cancers.

Activation of EGFR involves heterodimerization of EGFR with HER2. Our results showed that there was no correlation between EGFR and HER2 protein expression (P = 0.567), which might indicated that activated EGFR can form heterodimer not only with HER2, but also with other members of ErbB family.

Currently, many trials have assayed for EGFR gene amplification in order to identify patients that would benefit from anti-EGFR therapy. Patients with EGFR gene amplification have been connected to poor prognosis in HNSCC [49,50] and NSCLC [51]. However, patients with EGFR mutations have demonstrated an increased benefit as compared to patients having EGFR amplification [39]. EGFR gene mutations indicate sensitivity to gefitinib, and it was demonstrated that about 85% of patients with NSCLC who obtained benefit from gefitinib treatment were found to have mutations in exons 18 to 21 of the tyrosine kinase domain of the EGFR gene [51-54]. EGFR mutations in exon 19 (short in-frame deletions) or 21 L858R (point mutation) affect the adenosine triphosphate (ATP) pocket of the tyrosine kinase domain leading to the activation of 4-anilinoquinazoline compounds, which function to compete with ATP [53]. Anti-EGFR therapy can consequently lead to the downregulation of downstream signaling cascades, such as the PI3K/Akt, RAS/Erk, MAPK, and STAT pathways, responsible for cell proliferation and survival, resulting in the inhibition of cell proliferation and induction of cell apoptosis, respectively. The method for EGFR-mutations used in this study can detect the most common mutations of exons 19 and 21, but there are still other mutations of exons 19 and 21 that cannot be detected. Additionally, mutations of exons 18 and 20, which can harbor upto 15% of EGFR-mutations in lung cancer, cannot be analyzed in this way.

It is reported in some studies that EGFR gene mutations in the tyrosine kinase domain in patients with lung cancer are accompanied with a low increase in EGFR gene copy number [55,56]. However, in this study, EGFR gene mutations could be identified in only 2 out of 139 cases (1.4%) of the breast carcinoma samples, confirming that EGFR gene mutations are rare in Chinese patients. Further trials with large samples and/or different methods are highly recommended to be performed to validate the observations mentioned above.

## Conclusions

We observed that EGFR gene mutations were rare in breast carcinomas, but EGFR gene amplification was detected in about one third of the cases in this population. In this study, rare mutations in the EGFR gene in patients with breast cancer were detected, indicating that EGFR gene mutations are infrequent in this cohort of breast cancers. This suggested that EGFR mutation analysis is not useful as a screening test for sensitivity to anti-EGFR therapy for breast cancers. Nevertheless, further studies will be required to investigate whether EGFR gene copy number is a suitable screening test for EGFR targeted therapy.

## Abbreviations

ATP: adenosine triphosphate; CK5/6: cytokeratin 5/6; CRC: colorectal cancer; DAPI: 4': 6'-diamidino-2-phenylindole; EGFR: epidermal growth factor receptor; ER: estrogen receptor; FISH: fluorescence in-situ hybridization; HER2: human epidermal growth factor receptor-2; HNSCC: squamous cell carcinoma of the head and neck; MBC: metastatic breast cancer; MoAbs: monoclonal antibodies; NSCLC: non-small cell lung cancer; PI3K: phosphatidyl inositol 3'-kinase; PR: progesterone receptor; Real Time PCR: Real-time quantitative Polymerase Chain Reaction; SSC: saline sodium citrate buffer; TGF-α: transforming growth factor-alpha; TKI: tyrosine kinase inhibitors.

## Competing interests

The authors declare that they have no competing interests.

## Authors' contributions

WDW conceived and designed the study, WDW and NL performed the experiments, WDW and NL conducted the statistical analysis, and WDW and NL drafted the manuscript with substantial contributions from all authors. All authors read and approved the final manuscript.
